# Changes in Attitude to Waterpipe Tobacco Smoking among Youngsters in Eastern Province, Saudi Arabia: A Cross-Sectional Study

**DOI:** 10.31557/APJCP.2021.22.5.1443

**Published:** 2021-05

**Authors:** Royes Joseph, Dhfer Alshayban

**Affiliations:** *Department of Pharmacy Practice, College of Clinical Pharmacy, Imam Abdulrahman Bin Faisal University, Dammam, Saudi Arabia. *

**Keywords:** Smoking water pipes, intention-to-quit, intention-to-start, youngsters, Saudi Arabia

## Abstract

**Background:**

A growing number of epidemiological evidence suggests a significant increase in waterpipe tobacco smoking, and its potential to become a major public health concern in most Arabic countries, including Saudi Arabia.

**Methods:**

A cross-sectional study was carried out to assess the prevalence of intention to quit among ever users of waterpipe and intention to start among the never users. The study also investigated the barriers that may prevent users from quitting or trigger the nonusers to start waterpipe smoking. The study consisted of 464 university students from Eastern Province, Saudi Arabia.

**Results:**

One hundred and sixty-eight (36.2%) participants were responded that they had WTS at least one time in the past. Among the ever users of WTS, 120 (71.4%) participants had made an attempt or more to quit WTS in the past, 64 (38.1%) had made more than one attempt, and nearly two-third expressed the intention to quit WTS in the future. Forty (13.5%) out of 296 never-users expressed their intention to start WTS in the future. The study further showed that peer influence, social acceptance, and risk perception were significant predictors of intention to start or stop WTS among students.

**Conclusion:**

It is promising that substantial users have the intention to discontinue WTS, though a fraction of never users wish to try WTS in the future.

## Introduction

The water pipe device indirectly heats tobacco and produces smoke, which then passes through a column of water before being inhaled through the mouth using a pipe. It has different names, such as waterpipe, hookah, narghile, and arghile. A growing number of epidemiological evidence suggests a significant increase in waterpipe tobacco smoking (WTS), and its potential to become a major public health concern in most Arabic countries, including Saudi Arabia (Maziak et al., 2004a; Maziak et al., 2004b; Maziak, 2004). The increased use of WTS among the Saudi population may be attributed to different reasons, including cultural reasons and the misconception that smoking waterpipe is safe and not as harmful as smoking cigarettes. According to the World Health Organization (WHO) and other studies, WTS may be addictive as in other forms of tobacco use and may cause similar health risks that smoking cigarettes can cause, including lung cancer, respiratory illness, and low birth weight (Martinasek et al., 2011; Akl et al., 2011; WHO Study Group on Tobacco Product Regulation, 2015; Soule et al., 2015; Jawad et al., 2018; Qasim et al., 2019).

As part of tobacco-free initiatives, WHO has introduced MPOWER measures to promote government action on tobacco control (WHO EMRO, 2010). MPOWER consists of six evidence-informed and cost-effective interventions: Monitoring tobacco use, Protecting people from tobacco use through smoke-free policies, Offering cessation programs, health Warnings, Enforcing a ban on promotion, and make tobacco products less affordable by Raising taxes on them. In line with these measures, Saudi Arabia has enforced laws preventing smoking in most public places (Heydari et al., 2018; WHO, 2019). The country further ensured the availability of smoking cessation support through primary care health centers, availability of nicotine replacement therapy, Bupropion, and Varenicline through pharmacies with full health insurance coverage, and offered a toll-free telephone help-line to discuss cessation (Heydari et al., 2018; WHO, 2019). In the country, health warnings on tobacco packages are mandated by law, and a number of anti-tobacco mass media campaigns were aired (Heydari et al., 2018; WHO, 2019). As per the WHO report on global tobacco pandemic, Saudi Arabia has recently implemented the MPOWER measures at the moderate to best practice level except on the monitoring aspect (WHO, 2019). A significant reduction in the prevalence of tobacco use would be expected if the implementation of these measures at the highest level (WHO EMRO, 2010). The overall reduction is expected through the increase in smoking quit rate and reduction in smoking initiation rate.

The smoking-related beliefs and the intention to quit or start smoking among Saudi youth have been the focus of smoking cessation research. The smoking-related beliefs could be explained by a health belief model that is used to predict an action that people might take based on the belief to prevent, to screen for, or to control disease situations (Taylor et al., 2006; Balbach et al., 2006; Glanz et al., 2015). The first component of this model is the perception of susceptibility which refers to beliefs of users regarding the likelihood of having some negative consequences of this habit (Taylor et al., 2006; Balbach et al., 2006; Glanz et al., 2015). The second component is the perception of the seriousness of the health outcomes and the possible social of smoking that might impact the decision of people toward quitting or starting smoking (Balbach et al., 2006; Glanz et al., 2015). The third component of the model is the perception of the benefits of quitting or starting smoking. Furthermore, the perceptions of barriers and obstacles such as social barriers (i.e., having a close friend who smokes waterpipe) could lead the person to decide either to start or to quit waterpipe smoking (Balbach et al., 2006; Glanz et al., 2015). 

There is a lack of studies to investigate the factors related to intention to quit or start waterpipe smoking among smokers in Saudi Arabia. The present study has two objectives: 1) to determine the prevalence of intention to quit among the WTS users and the intention to start among the nonusers; 2) to examine socio-demographic characteristics, smoking-related beliefs as predictors of intention to quit and intention to start WTS among Saudi university students. The impact of our study was that it might help to know in-depth the social, cultural, and other factors around smoking waterpipe, in order to overcome all the barriers that may prevent users from quitting or trigger nonusers to start WTS.

## Materials and Methods

*Study design and sample*

We conducted a questionnaire-based cross-sectional study among university students. The study sample was selected from various colleges of a leading public university in the Eastern province of Saudi Arabia. The university has more than 45,000 registered students over 21 colleges that spread over the Eastern Province, Saudi Arabia. The study sample comprised of male and female undergraduate students aged below 18 years under health, engineering, and arts and science stream of studies. A minimum sample size of 323 participants was required to estimate a prevalence of 30% with 5% absolute precision and a 95% confidence level. Therefore we targetted a sample of 500 students with the expectation of incomplete data from some students. A group of final-year students had been selected and trained for data collection using a questionnaire. The data collectors approached students at public places, such as the atrium, cafeteria, library, and other open areas, within the university in order to collect the data. The purpose of the study was explained, and consent for participation was obtained from the participating students. The participants were requested to fill the questionnaire with minimal support from the data collectors. Ethical approval was obtained from the Institutional Ethics Committee, College of Clinical Pharmacy, Imam Abdulrahman Bin Faisal University.

*Questionnaire*

The questionnaire has four sections: socio-demographic information of participants, smoking status and family history, the perceptions on WTS, and the information on the willingness to quit or try WTS in the future. The first three sections were adapted from literature on WTS use (Primack et al., 2008).

*Socio-demographic details*

Data on age, gender, study stream, marital status, area of residence (urban/rural), and monthly family income of participants were collected.

*Smoking status and family history*

Participants were asked if they had smoked cigarettes, e-cigarettes, and waterpipe in the past ever in addition to a question on their 30-days use. The frequency of WTS use among friends and family members were also obtained.

*Perceptions on WTS*

The questionnaire contained eight items to assess risk-perception about WTS, knowledge on the hazards of WTS, and the normative belief on the social acceptance of WTS. The beliefs about the addictive and harmful effects of WTS compared to cigarettes were rated on a five-point scale. The lowest score of ‘0’ indicated ‘WTS is much less addictive/harmful than cigarettes’, and the highest score of 4 indicated ‘WTS is much more addictive/harmful than cigarettes’. The overall risk-perception was assessed using the sum of the two items with a lower total score indicates the higher the false perception. The knowledge on the hazards of WTS was assessed by asking the participants to rate five statements about WTS. The statements were 1) water filters the toxic elements of WTS, 2) WTS is free of tar, 3) WTS is free of nicotine, 4) WTS is free of carbon monoxide, and 5) no increased risk of cardiovascular diseases (CVD) due to WTS. The participants rated the statements on a five-point Likert scale ranging ‘strongly agree (score 0)’ to ‘strongly disagree (score 4)’. A total score of these five items were calculated as a measure of knowledge on hazards of WTS with a lower score indicates lower the knowledge. In order to assess the normative belief on social acceptance of WTS, participants were requested to rate their belief on acceptance of WTS in the society on a four-point scale ranging ‘not acceptable’ to ‘very acceptable’.

*Intention to quit or start in the future*

Participants who had used WTS in the past were asked about their previous attempt to quit, previous use of cessation mediations/programs, and their willingness to quit in the future. Their self-confidence with the quit decision was also rated. Participants who never used WTS in the past were also asked their intention to try WTS in the future.

*Data analysis*

‘Ever user’ of WTS was a participant who used WTS in the past, while ‘never user’ of WTS was a participant who never used WTS in the past. We estimated the prevalence of intention to quit among the ever users and intention to try among never users. For the purpose of analysis, the total risk perception score and knowledge score were categorized into three levels using tertiles: categories with high scores (low favored for WTS), moderate scores, and low scores (highly favored for WTS). Further, categories of individual items were collapsed into three categories. 

Data were summarized using frequencies and percentages. A Chi-square test for association was used to identify the predictors of intention to quit and start WTS in the future. A p-value of less than 0.05 was considered statistically significant. Analyses were carried out using SPSS Statistics version 24.0.

## Results

The study sample consisted of 464 participants, of which 268 (57.8%) were males. The description of the participants was detailed in Table 1. 

*Ever users of WTS*

**Figure 1:**
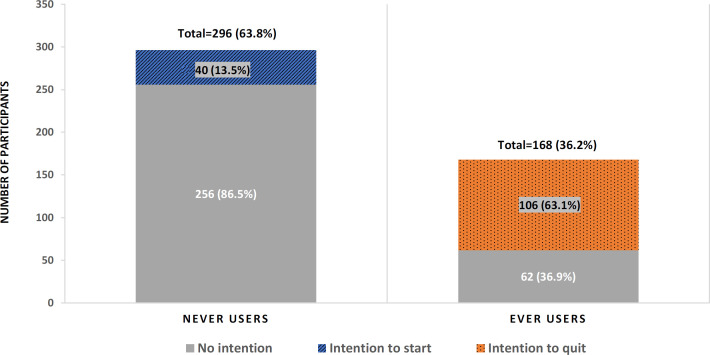
Proportion of Participants Showed Intention to Start and Intention to Quit Waterpipe Smoking

One hundred and sixty-eight (36.2%) participants were responded that they had WTS at least one time in the past (Figure 1). Column 5 in Table 2 reports the prevalence of ever use of WTS by participants’ characteristics. Significantly higher WTS prevalence of 43.3% (116/268), 41.3% (124/300), 72.5% (74/102), and 52.5% (106/202) were reported among male gender, students of health cluster, current users of cigarettes and e-cigarettes, and students who had family history of occasional/frequent use of WTS, respectively, compared to their corresponding counterparts (p <0.05). Further, as shown in Table 3 (column 5), significantly lower WTS prevalence of 28.6% (40/140), 25.0% (38/152), 22.2% (28/126), 23.4% (36/154), 24.4% (44/180), 26.0% (54/208), 26.9% (70/260), and 17.8% (26/146) among students who believed ‘waterpipe is more harmful than cigarettes’, ‘waterpipe is more addictive than cigarettes’, ‘water does not filter toxins’, ‘STS contains tar’, ‘STS contains carbon monoxide’, ‘increased risk of CVD’, and ‘STS is not socially acceptable’, respectively, compared to their corresponding counterparts (p <0.05). 

*Intention to start WTS in the future among never-users*

There were 40 (13.5%) out of 296 never-users were expressed their intention to start WTS in the future (Figure 1). The proportion of participants who intend to start WTS in the future among never-users was given in Tables 2 and 3 (columns 3 and 4). The proportion among males (18.4%), students of Arts and Science cluster (29.0%), current users of cigarettes (57.1%) and e-cigarettes (55.6%), students with family history of frequent use of WTS (26.9%) were significantly higher than the proportions in their corresponding counterparts (p <0.05). The proportion was lower, but not statistically significant, among students with high-risk perception (6.8%) and high knowledge score (10.2%) compared to students with low-risk perception (17.9%) and low knowledge score (18.2%), respectively. Importantly, 23.1% (6/26) of students who think WTS is well accepted had expressed their intention to use WTS in the future; while it was only 6.7% (8/120) among students who think WTS is not accepted socially.

*Previous quitting attempt and intention to quit WTS in the future among ever users*

Among the ever users of WTS, 120 (71.4%) participants had made an attempt or more to quit WTS in the past; 64 (38.1%) had made more than one attempt. More than half of users (94/168) were aware of cessation services for WTS. However, only 14.3% (24/168) had used the service: Bupropion was used by four participants, Varenicline or NicoDerm CQ was used by 16 participants, and other medications were used by another four participants.

Among the ever users of WTS, nearly two-thirds (106/168) expressed the intention to quit WTS in the future (Figure 1). The proportion by participants’ characteristics was given in Tables 2 and 3 (columns 6 and 7). The proportion was significantly higher among males (74.1%), urban students (67.6%), e-cigarettes non-smokers (68.4%), and students who did not have the family history of WTS use (94.1%), compared to their corresponding counterparts. Further, the proportion was lower among the students who believe waterpipe is less harmful (46.3%), waterpipe is less addictive (56.3%), water filter toxins substantially (46.2%), and waterpipe is not associated with CVD (51.4%). Importantly, less than one-half of participants with a low risk-perception score (43.3%) and participants who believed that shish is very socially acceptable (30.0%) were expressed intention to quit WTS in the future. 

**Table 1:** Socio-Demographics Characteristics of Participants (N=464)

Factors	Total n (%)
Gender	
Male	268 (57.8%)
Female	196 (42.2%)
Area of study	
Health	300 (64.7%)
Engineering	78 (16.8%)
Arts, Science and Management	86 (18.5%)
Locality	
Urban	378 (81.5%)
Rural	86 (18.5%)
Marital status	
Single	436 (94%)
Married	28 (6%)
Family monthly income	
Less than 5,000 SR	74 (15.9%)
5,000-15,000	142 (30.6%)
16,000-25,000	154 (33.2%)
Greater than 25,000	94 (20.3%)

**Table 2:** Socio-Demographics and Cigarettes Smoking among Ever and Never Users of Waterpipe Tobacco Smoking

Variables		Never users (N=296)			Ever users (N=168)		
		Total	Intention to start (N=40)		Total	Intention to quit (N=106)	
		n (%)	n (%)	p-value	n (%)	n (%)	p-value
Gender							
	Male	152 (56.7)	28 (18.4)	0.011*	116 (43.3)*	86 (74.1)	<0.001*
	Female	144 (73.5)	12 (8.3)		52 (26.5)	20 (38.5)	
Area of study							
	Health	176 (58.7)	18 (10.2)	<0.001*	124 (41.3)*	72 (58.1)	0.072
	Engineering	58 (74.4)	4 (6.9)		20 (25.6)	16 (80.0)	
	Arts & Science	62 (72.1)	18 (29.0)		24 (27.9)	18 (75.0)	
Locality							
	Urban	236 (62.4)	32 (13.6)	0.964	142 (37.6)	96 (67.6)	0.005*
	Rural	60 (69.8)	8 (13.3)		26 (30.2)	10 (38.5)	
Marital status							
	Single	282 (64.7)	38 (13.5)	0.931	154 (35.3)	100 (64.9)	0.101
	Married	14 (50)	2 (14.3)		14 (50.0)	6 (42.9)	
Family monthly income							
	Less than 5000 SR	44 (59.5)	4 (9.1)	0.375	30 (40.5)	22 (73.3)	0.196
	5,000-15,000	100 (70.4)	18 (18)		42 (29.6)	30 (71.4)	
	16,000-25,000	92 (59.7)	12 (13)		62 (40.3)	34 (54.8)	
	Greater than 25,000	60 (63.8)	6 (10)		34 (36.2)	20 (58.8)	
Cigarettes used within 30 days							
	No	268 (74.0)	24 (9.0)	<0.001*	94 (26.0)*	62 (66.0)	0.386
	Yes	28 (27.5)	16 (57.1)		74 (72.5)	44 (59.5)	
E-Cigarettes used with in 30 days							
	No	278 (70.9)	30 (10.8)	<0.001*	114 (29.1)*	78 (68.4)	0.038*
	Yes	18 (25.0)	10 (55.6)		54 (75.0)	28 (51.9)	
Use of Waterpipe by family							
	Never used	134 (79.8)	16 (11.9)	0.018*	34 (20.2)*	32 (94.1)	<0.001*
	Rarely used	66 (70.2)	6 (9.1)		28 (29.8)	12 (42.9)	
	Occasionally used	44 (48.9)	4 (9.1)		46 (51.1)	26 (56.5)	
	Frequently used	52 (46.4)	14 (26.9)		60 (53.6)	36 (60.0)	

*p-value<0.05

**Table 3:** Risk-Perception, Knowledge and Normative Beliefs among Ever and Never Users of Waterpipe Tobacco Smoking

Variables		Never users (N=296)			Ever users (N=168)		
		Total	Intention to start (N=40)		Total	Intention to quit (N=106)	
		n (%)	n (%)	p-value	n (%)	n (%)	p-value
Belief about harmfulness							
	Waterpipe is more harmful	100 (71.4)	8 (8.0)	0.139	40 (28.6)*	34 (85.0)	<0.001*
	Waterpipe is equally harmful	100 (68.5)	16 (16.0)		46 (31.5)	34 (73.9)	
	Waterpipe is less harmful	96 (53.9)	16 (16.7)		82 (46.1)	38 (46.3)	
Belief about addictiveness							
	Waterpipe is more Addictive	114 (75.0)	12 (10.5)	0.161	38 (25.0)*	26 (68.4)	0.024*
	Waterpipe is equally Addictive	74 (68.5)	8 (10.8)		34 (31.5)	26 (76.5)	
	Waterpipe is less Addictive	108 (52.9)	20 (18.5)		96 (47.1)	54 (56.3)	
Risk-perception score (overall)							
	Low (highly favoured for waterpipe)	56 (48.3)	10 (17.9)	0.084	60 (51.7)*	26 (43.3)	<0.001*
	Moderate	152 (63.9)	24 (15.8)		86 (36.1)	64 (74.4)	
	High (Less favoured for waterpipe)	88 (80.0)	6 (6.8)		22 (20.0)	16 (72.7)	
Belief about water filtering of toxins							
	Completely‎/Substantially	68 (56.7)	10 (14.7)	0.151	52 (43.3)*	24 (46.2)	0.007*
	Moderately	130 (59.6)	22 (16.9)		88 (40.4)	64 (72.7)	
	Slightly‎/Nothing	98 (77.8)	8 (8.2)		28 (22.2)	18 (64.3)	
Belief about absence of tar							
	Agree	138 (57.5)	26 (18.8)	0.031*	102 (42.5)*	58 (56.9)	0.074
	Neutral	40 (57.1)	2 (5.0)		30 (42.9)	20 (66.7)	
	Disagree	118 (76.6)	12 (10.2)		36 (23.4)	28 (77.8)	
Belief about absence of nicotine							
	Agree	118 (57.8)	20 (16.9)	0.298	86 (42.2)*	56 (65.1)	0.297
	Neutral	42 (52.5)	6 (14.3)		38 (47.5)	20 (52.6)	
	Disagree	136 (75.6)	14 (10.3)		44 (24.4)	30 (68.2)	
Belief about absence of CO							
	Agree	114 (54.3)	18 (15.8)	0.619	96 (45.7)*	62 (64.6)	0.317
	Neutral	28 (60.9)	4 (14.3)		18 (39.1)	11 (61.1)	
	Disagree	154 (74.0)	18 (11.7)		54 (26.0)	38 (70.4)	
Belief about increased risk of CVD							
	Agree	190 (73.1)	24 (12.6)	0.764	70 (26.9)*	54 (77.1)	0.005*
	Neutral	48 (66.7)	8 (16.7)		24 (33.3)	14 (58.3)	
	Disagree	58 (43.9)	8 (13.8)		74 (56.1)	38 (51.4)	
Knowledge score (overall)							
	Low (highly favoured for waterpipe)	66 (47.1)	12 (18.2)	0.299	74 (52.9)*	40 (54.1)	0.093
	Moderate	112 (65.9)	16 (14.3)		58 (34.1)	40 (69.0)	
	High (Less favoured for waterpipe)	118 (76.6)	12 (10.2)		36 (23.4)	26 (72.2)	
Normative belief (Social acceptance)							
	Not acceptable	120 (82.2)	8 (6.7)	0.021*	26 (17.8)*	24 (92.3)	<0.001*
	Somewhat acceptable	104 (68.4)	20 (19.2)		48 (31.6)	34 (70.8)	
	Moderately acceptable	46 (46.0)	6 (13.0)		54 (54.0)	36 (66.7)	
	Very acceptable	26 (39.4)	6 (23.1)		40 (60.6)	12 (30.0)	

*p-value<0.05

## Discussion

The present study investigated the prevalence of the previous attempt to quit, and intention quit WTS among waterpipe ever users and the prevalence of intention to start WTS among the never users in the Eastern Province, Saudi Arabia. Further to aid in developing effective strategies for discouraging the use of WTS among youngsters, the study further investigated the differences in their characteristics, including socio-demographics, knowledge, and perception about WTS, between users with and without intention to quit WTS and nonusers with and without intention to start WTS. The study population was undergraduate students. The study shows that more than one-third (36%) of participants were used WTS at least once in the past with a higher proportion among males than in females. A previous publication was reported that the past 30-day prevalence was 23% with a narrow gender gap (Alshayban and Joseph, 2019). The previous attempt or the intention to quit may explain the gender difference in proportion. Availability, affordability, and attractiveness of waterpipe may be the key reasons for the higher prevalence of WTS use among youngsters (Maziak et al., 2004c). We found that 63% of ever users expressed the intention to quit, and 14% of never users expressed their intention to start in the future. A study from Qatar reported that more than half of waterpipe users admitted to intending to quit the use (Jaam et al., 2016). A similar proportion of intention to quit smoking was reported among cigarette smokers in Saudi Arabia (Al-Zalabani et al., 2015; Almogbel et al., 2016). Although it is promising that substantial users have the intention to discontinue WTS, still a number of never-users expressed their intention to try WTS in the future. Thus, it is quite important to introduce a more pervasive restriction on WTS in public places and make it less attractive in order to help the students to refrain from WTS. Health warning labels are found to be effective in encouraging students to quit WTS (Darawad et al., 2019).

Previous studies have expressed concern over awareness about WTS cessation techniques among the general public and particularly, among the practicing physicians (Jradi, 2017; Romani et al., 2020). The present study reported that nearly three-fourth of ever users had made an attempt or more to quit WTS in the past. Further, only 14% used a cessation service for WTS, though half of WTS users were aware of such cessation services. More importantly, a systematic review highlighted the lack of evidence on the effectiveness of interventions targeting prevention and cessation of WTS and a need for higher quality effective clinical and behavioral interventions (Jawad et al., 2016).

The study found that more males expressed the intention to quit WTS among the users, and in contradictory, more males also expressed the intention start to start WTS in the future among never users. Thus, the imbalance in the proportions leads to a narrow gender gap on the prevalence of past one-month use of WTS, as reported in our previous study. Our finding was in line with a previous study that assessed the willingness to quit cigarette smoking among youngsters, and the study observed that a substantially higher proportion of males preferred to quit smoking than females (Al-Zalabani et al., 2015). The higher proportions among the male students may be linked to their confidence to stop WTS at any time point.

We observed, as reported in Table 1, that nearly three-fourth of current users of cigarette or e-cigarette were ever users of WTS in comparison with a quarter among the nonusers of cigarette or e-cigarette. In line with this finding, the study further observed that the willingness to quit and intention start WTS is directly associated with the use of other forms of tobacco. In this study, a lower proportion of intent quit and higher intent to start were reported among the cigarette or e-cigarette users. It strongly indicates the popularity and acceptance of WTS among the users of other-form of tobacco (Alshayban and Joseph, 2019). 

Smoking behavior of family members and peers is strongly associated with the initiation of WTS among youngsters and women (Baheiraei et al., 2015). A similar association was observed in our study. The study found that WTS use was higher among students with a family history of frequent use of WTS. Importantly, the intent to start rate and the intent to quit rate was higher among the students had the family history of use and did not have the family history, respectively. The smoking behavior among the closed ones may contribute to the level of perceived social acceptance of WTS. Among the never users, only 7% of students who think WTS is not socially acceptable were showed intention to start WTS while it was 23% among the students who think WTS is well accepted. Among the ever users, 92% of students who think WTS is not socially acceptable were showed intention to quit WTS while it was only 30% among the students who think WTS is well accepted. This indicates the perception of social acceptance of WTS has a significant effect on preventing the participants for refraining from WTS. Therefore, appropriate measures need to be taken for discouraging the use of waterpipe during social gatherings in order to prevent from perceiving WTS as a kind of entertainment and is considered acceptable in society.

Previous studies have demonstrated that the higher prevalence of WTS use is associated with a high level of perceived lack of harmful and addictive effects of WTS among university students (Abu-Rmeileh et al., 2018). In the current study, more than one-third of participants were misconceived that waterpipe is less harmful and less addictive than cigarettes, and a higher proportion of them were WTS users. We observed that the willingness to quit among the users is significantly associated with the misconception. In overall, three-quarters of users with low-risk perception were expressed willingness to quit while only 43% intended to quit among the students with high-risk perception. Among the nonusers of WTS, a similar difference in the proportion of students who plan to initiate WTS between the levels of risk perception, though it was not statistically significant. The findings indicate that targeting beliefs on the harmfulness and addictiveness may influence the willingness to quit among users and desire to try among nonusers. Mandated waterpipe tobacco package warning may result an increase of awareness of the health effects of WTS (King et al., 2019), and thus increase in the level of risk perception (Salloum et al., 2017).

It was found that students who think WTS is free of tar, nicotine, or carbon monoxide are at higher risk of start using WTS compared to those who think otherwise. Alternatively, the students who think the WTS is free of the hazardous contents are less likely to stop WTS among users compared to those who think otherwise. The users who think that WTS has increased risk of CVD are more prone to quit compared to the users who think otherwise. In overall, though not statistically significant, the desire to try WTS was reported by 18% of the students with knowledge score that highly favored for WTS in comparison with 10% among the students with knowledge score that less favored for WTS. Similarly, 72% of students with knowledge scores that less favored for WTS were showed intention to quit WTS in comparison with 54% among the students with knowledge scores that highly favored for WTS. Thus, it is important to educate and increase the awareness of students and public on the impact of WTS on health. The textual and pictorial warning on waterpipe tobacco boxes is found to be effective in increasing motivation and intention to stop WTS (Hallit et al., 2019).

This is the first study in the Kingdom of Saudi Arabia to investigate the willingness to quit among WTS users and desire to try WTS among nonusers and its association with the risk perception and knowledge on the health effects of WTS. This was a cross-sectional study, and thus causal association could not be established. Prospective studies are required to show how risk perception and knowledge on the health effects of WTS influence the cessation among WTS users and desire to try WTS among nonusers. Though the study sample selection from different colleges ensured participation from the diverse socio-economic background, the majority of participants were from the Eastern Province, Saudi Arabia. 

In conclusion, the study identified the impact of peer influence, social acceptance, and risk perception on behavioral intention to start or stop WTS among students. It is promising that substantial users have the intention to discontinue WTS, though a fraction of never users wish to try WTS in the future. Further actions such as introducing more pervasive restrictions on WTS in public places, make it less attractive, mandating health warning on waterpipe tobacco packages, and awareness campaign on the effectiveness of interventions targeting prevention and cessation of WTS is warranted in order to help the students to refrain from WTS. 

## Author Contribution Statement

DA conceived the idea, and later developed as a study in consultation with RJ. DA and RJ equally contributed to the study design. DA was responsible for the data collection. RJ was responsible for the data management and statistical analyses. DA drafted the manuscript. Both authors contributed to further revisions to the draft. All authors have read and approved the final manuscript.
